# Comparative Study Between Cognitive Phenotypes of Myalgic Encephalomyelitis/Chronic Fatigue Syndrome and Multiple Sclerosis

**DOI:** 10.3390/diagnostics15040487

**Published:** 2025-02-17

**Authors:** Mehdi Aoun Sebaiti, Nadia Oubaya, Yannick Gounden, Chloé Samson, Emmanuele Lechapt, Abir Wahab, Alain Creange, Mathieu Hainselin, François-Jérôme Authier

**Affiliations:** 1 CRP-CPO, UR UPJV 7273, Université de Picardie Jules Verne, F-80025 Amiens, France; yannick.gounden@u-picardie.fr (Y.G.); mathieu.hainselin@u-picardie.fr (M.H.); 2 INSERM, IMRB, Université Paris Est Créteil, F-94010 Créteil, Francefrancois-jerome.authier@aphp.fr (F.-J.A.); 3 Néocortex (Spécialistes de la Neuropsychologie), F-94100 Saint-Maur-des-Fossés, France; 4 Département de Santé Publique, AP-HP, Hôpital Henri-Mondor, F-94010 Créteil, France; 5 AP-HP, Hôpital René Muret, F-93270 Sevran, France; chloesamsonb@gmail.com; 6 Département de Pathologie, AP-HP, Hôpital Henri Mondor, F-94010 Créteil, France; emmanuele.lechapt@aphp.fr; 7 Service de Neurologie, AP-HP, Hôpital Henri Mondor, F-94010 Créteil, France; abir.wahab@aphp.fr (A.W.); alain.creange@aphp.fr (A.C.); 8 UF Centre Expert de Pathologie Neuromusculaire, AP-HP, Hôpital Henri Mondor, F-94010 Créteil, France

**Keywords:** neuropsychological battery, cognition, disorders, memory, reading speed, specific profile

## Abstract

**Objective**: Cognitive impairments are one of the most common and disabling symptoms associated with myalgic encephalomyelitis/chronic fatigue syndrome (ME/CFS). Here, we address the possibility of a specific cognitive profile inherent to ME/CFS. Due to the occurrence of cognitive deficits, fatigue, and pain in both pathologies, multiple sclerosis (MS) is a relevant comparison model. For this purpose, we carried out a comparative study between cognitive profiles of patients with ME/CFS and patients suffering from MS. **Methods**: In total, 40 ME/CFS and 40 MS patients were included. A complete screening of all cognitive functions was carried out through an extensive battery of tests routinely used in clinical practice. **Results**: ME/CFS and MS patients showed deficits in episodic memory retrieval, visual selective attention and reading speed. ME/CFS patients also elicited a lower level of performance than MS patients regarding consolidation. For both groups, levels of performance on these cognitive tests did not correlate with levels of fatigue, pain, and depression. **Conclusions**: This study highlighted both similarities and differences in the cognitive profiles of ME/CFS and MS patients. While both groups exhibited deficits in episodic memory retrieval, visual selective attention, and reading speed, ME/CFS patients showed distinct impairment in consolidation processes. These cognitive deficits were not correlated with fatigue, pain, or depression, reinforcing the hypothesis of intrinsic cognitive dysfunction in ME/CFS. These findings define a specific cognitive phenotype for ME/CFS, which could improve diagnostic accuracy and therapeutic strategies. Future research, particularly in functional imaging, may elucidate the neurobiological mechanisms underlying these impairments.

## 1. Introduction

ME/CFS is characterized by the presence of severe, unexplained fatigue that persists for a minimum of six months. This fatigue is accompanied by central nervous system and immune system disorders, leading to a significant reduction in occupational or leisure activities [1]. In healthy individuals, fatigue is usually proportional to the effort expended or its duration, and a swift recovery is typically observed, with recurrence to the same extent with the same amount of effort or duration. In myalgic encephalomyelitis/chronic fatigue syndrome (ME/CFS), the pathological threshold of fatigability is typically reached with minimal physical or mental exertion, resulting in a reduced ability to engage in the same activity on the same day or several days later. The condition is characterized by a constellation of symptoms, including self-reported memory and attention problems, tender lymph nodes, muscle pain, multi-joint pain, sore throat, headaches, un-refreshing sleep, and post-exertional malaise. These clinical features are employed as diagnostic criteria. To date, as many as 20 case definitions have been advanced [2], and those from Fukuda [3] are the most commonly used [4]. The 2011 International Consensus Criteria are a set of guidelines that provide a framework for the diagnosis and management of medical conditions. These criteria were developed through a consensus process involving experts from various fields of medicine, and they are intended to ensure that patients receive consistent and high-quality care [5]. In comparison to the CDC criteria, the new classification system incorporates three significant modifications. First, the waiting period of six months before diagnosis is no longer required. Second, post-exertional malaise, characterized by an atypical intolerance to exercise with a recovery time that exceeds normal expectations, has been incorporated as a mandatory component of the classification. Third, the identification of symptoms associated with chronic fatigue has been divided into three distinct groups: (a) indicators of an impairment of the nervous system, (b) indicators of an impairment of the immune, gastrointestinal, and genitourinary systems, and (c) indicators of an impairment of energy production/distribution systems. The presence of indicators indicative of impairment to the nervous system is observed. Concurrently, indicators of impairment to the immune, gastrointestinal, and genitourinary systems are also present. Furthermore, indicators of impairment to the energy production and distribution systems are identified. These criteria allow for a more precise case definition and the stratification of patients. Recently, the Institute of Medicine (IOM) [6] issued a proposal to rename ME/CFS as systemic exertion intolerance disease (SEID), which is a significant development in the field. This proposed change aims to encapsulate the central elements of the disease, offering a more comprehensive and descriptive alternative to the existing nomenclature. The present report focuses on the adverse effects that physical, cognitive, or emotional exertion can have on patients with this condition. It acknowledges that this is a complex and severe disorder for which specific causes are not yet known. In the present study, the ME/CFS denomination will be retained, as it is more commonly used.

Cognitive manifestations represent one of the most prevalent and debilitating symptoms associated with ME/CFS [7]. A significant proportion of patients, amounting to 89%, exhibited symptoms indicative of memory and concentration difficulties [8]. Since the 1980s, clinicians have administered formal neurocognitive assessment tests to patients with ME/CFS. A comprehensive review of 764 studies published between 1988 and 2019 revealed that the neuropsychological profile characteristic of ME/CFS is typified by a diminished efficiency of visuospatial immediate memory, an augmented reaction time, and a reduction in reading speed and graphomotricity. This analysis also revealed difficulties in several processes inherent in episodic verbal memory (storage, retrieval, and recognition) and visual memory (recovery) and a low efficiency in attentional abilities in terms of both auditory and visual inputs. Conversely, the study found that executive functions appeared to be largely unaffected, and instrumental functions demonstrated consistent preservation [9].

The diagnosis of ME/CFS requires ruling out any psychiatric or neurological condition that could explain fatigue, foremost among which is multiple sclerosis (MS) [10], which is typically observed in the same age group as ME/CFS patients. Unlike ME/CFS, MS typically includes brain MRI changes, which are part of the diagnostic criteria [11,12,13] (see the Supplementary Materials). Most patients initially present with the relapsing–remitting form [14], but the condition usually progresses over time [15]. Cognitive dysfunctions are present in 40 to 70% of MS patients [15,16], often early on in the course of disease [17]. Their identification is essential, given their consequences on autonomy, quality of life, professional life, and the social sphere [16]. They typically manifest by ideomotor slowing down and attentional and memory deficits [15,16]. In everyday life, these cognitive difficulties result in a general slowdown and difficulties in decision making, planning, and memorizing [18]. Episodic memory is impaired in both verbal and visual modalities [19,20,21], as well as working memory [16,20] and attentional abilities [22], including sustained and divided attention. Many patients present with impaired executive functions [22], as seen in their difficulties in cognitive flexibility, cognitive inhibition, planning, abstraction [16], and information generation [16].

The presence of both cognitive complaints and unexplained fatigue in ME/CFS often leads physicians to first suspect MS. Although the profiles of cognitive dysfunctions seem apparently comparable in ME/CFS and MS [23], it was reported that MS patients performed poorer than ME/CFS patients in verbal episodic memory, attention, working memory, mental flexibility, and generation of information tasks [24]. These findings are in keeping with studies showing that, in MS, cognitive impairment relates to the volume of lesions, which are mainly located in white matter and, therefore, can disrupt neural networks [15,16,25]. Conversely, in ME/CFS, no obvious brain injuries viewed using standard diagnostic imaging may explain cognitive impairments, and cognitive complaints are usually trivialized and regarded as a simple functional consequence of chronic fatigue [9]. In routine practice, we observed that cognitive dysfunction notably contributes to the chronic disability seen in ME/CFS. Moreover, it seems comparable in terms of intensity and profile to that observed in MS patients. To our knowledge, whether the cognitive profiles in MS and ME/CFS differ or not has not been explicitly investigated by cognitive tests. To address this issue, we conducted a retrospective study analyzing the neuropsychological data obtained in 40 ME/CFS and 40 MS patients, all having been evaluated in the same center and under the same modalities.

This study would allow a better understanding of the origins of the cognitive disorders inherent in ME/CFS and the development of adapted therapeutic strategies to improve the daily lives of patients.

## 2. Materials and Methods

The study was conducted in accordance with the Helsinki Declaration. We performed a descriptive, retrospective, comparative, and single-center study, carried out at Henri Mondor Hospital (Department of Neurology and Neuromuscular Pathology Expert Center), Créteil, France. The present research is based entirely on the analysis of data from clinical neuropsychological assessments carried out in the context of care. This study was approved (Approval No. 21-774) by the INSERM Institutional Review Board (IRB00003888, IORG0003254, and FWA00005831). Informed consent was obtained following an information and non-opposition process. Patients were verbally informed at the end of their neuropsychological assessment about the potential use of their clinical data for research purposes. Additionally, a written note of non-opposition was systematically included in the letters sent to all patients, allowing them to exercise their right to refuse data usage at any time (see Supplementary Materials).

### 2.1. Patients

Out of the 226 ME/CFS patients meeting the Fukuda/CDC1994 criteria [3], we selected 40 consecutive patients who reported cognitive difficulties and underwent a standardized and complete neuropsychological evaluation. Inclusion criteria for ME/CFS patients included a confirmed diagnosis according to the Fukuda/CDC1994 criteria, the presence of self-reported cognitive complaints, and the ability to complete a full neuropsychological assessment. Patients were not included if they did not report cognitive difficulties or had received a diagnosis outside the defined age range (18–65 years). Exclusion criteria included the presence of neurological or psychiatric disorders, like a history of head trauma, stroke, or neurodegenerative disease, that could significantly impact cognitive performance.

During the same period, 162 MS patients underwent neuropsychological evaluation. Among them, 15 patients were excluded due to neurological comorbidities, 12 due to unconfirmed MS diagnoses, and 61 because their neuropsychological assessments were incomplete or not comparable to those performed in ME/CFS patients. This left 74 remaining MS patients, from whom we excluded the 3 oldest patients to ensure an age-matched cohort of 71 MS patients.

To further refine the selection and minimize confounding factors, we applied additional inclusion and exclusion criteria to the MS group. Patients were included if they had a confirmed diagnosis of relapsing–remitting MS (RRMS) according to the revised McDonald criteria (2017) [12], reported cognitive difficulties, and had not received immunomodulating treatment at the time of evaluation, given the potential effects of treatments on cognitive performance [26]. Patients not included were those with progressive forms of MS, due to the differences in profile between the forms of MS [15,16,25], or those unable to complete the cognitive assessment. Exclusion criteria included the presence of neurological or psychiatric comorbidities, like a history of traumatic brain injury or other neurodegenerative disorders, and any MRI findings inconsistent with typical MS-related lesions.

This rigorous selection process ensured that both groups (ME/CFS and MS) were methodologically comparable, reducing the influence of confounding variables, such as disease heterogeneity or treatment effects, on cognitive performance. The application of clearly defined inclusion, non-inclusion, and exclusion criteria strengthened the validity of our comparisons and enhanced the interpretability of the results.

For all patients, the socio-educational level was graded as follows: level #1, illiterate; level #2, can read, write, and count (1st–2nd grades of primary/elementary school); level #3, primary/elementary school completed; level #4, middle/junior high school completed; level #5, 11th grade (junior year) of high/senior high school completed; level #6, 12th grade (senior year) of high/senior high school completed; level #7, university/college.

Mood treatments for ME/CFS patients included pregabalin (*n* = 5), duloxetin (*n* = 9), and amitriptylin (*n* = 8); for MS, this included paroxetin (*n* = 8), escitalopram (*n* = 5), and duloxetin (*n* = 2). Mood treatments in MS patients included selective serotonin reuptake inhibitors (*n* = 12) and serotonin and norepinephrine reuptake inhibitors (*n* = 9). The delays (years; mean ± SD) elapsed between the onset of disease and neuropsychological impairment in ME/CFS and MS patients were 13.1 ± 5.8 and 10.8 ± 8.3, respectively (NS; Table 1).

There was no significant difference between the two groups of patients with regard to age, educational level, and gender. The mean age (mean ± SD) of ME/CFS and MS patients was 46.9 ± 12.9 and 45.7 ± 11.6, respectively. The socio-educational level (SCL) of the participants had a median of 6 in both groups, with values ranging from 3 to 7 for the ME/CFS group and from 2 to 7 for the MS group. The sex ratios (female/male) of ME/CFS and MS patients were 22/18 and 27/13, respectively (Table 1).

### 2.2. Neuropsychological Assessment

The cognitive screening (Table 2) included tests that are conventionally utilized in neuropsychological evaluation. These tests were selected to reduce the evaluation’s duration while ensuring a reliable phenotyping of patients. The neuropsychological assessment encompassed the evaluation of short- and long-term memory in both verbal and visual modalities, attention, processing speed, executive functions, and instrumental functions. The evaluation of immediate memory was conducted using the forward digit span in verbal modality (WAIS IV, Pearson, Paris, France) [27]. Furthermore, consideration must be given to Corsi’s blocks (Developed by Philip Michael Corsi, McGill University, Montreal, QC, Canada) [28] for the visual one. The evaluation of working memory was carried out by a backward condition of digit span and Corsi’s blocks tests. The French version of the Free and Cued Selective Reminding Test (FCSRT) (Grober & Buschke adaptation for French populations, Paris, France) was used to evaluate verbal long-term memory [29]. The present study examined the three stages of the information processing model: encoding, storage, and retrieval [30]. Subsequently, the phases of recognition and consolidation of the memory trace were incorporated [29]. The visual modality of long-term memory was incidentally evaluated using the Rey-Osterrieth Complex Figure (Original test by Rey & Osterrieth, commonly used in neuropsychology) [31] (recall phase). The evaluation of visual selective attention was conducted using Zazzo’s cancellation test [32]. The evaluation of processing speed was conducted using the Trail Making Test A (TMT A) and the Stroop test (both used following the GREFEX recommendations, France) [33]. The TMT facilitates the consideration of the velocity of an act that integrates visual perception and motor gestures. In contrast, the Stroop test does not involve motor gestures, thereby allowing for the estimation of reading speed (particularly in the words condition) without the potential bias of graphomotor slowing down. The screening procedure entailed the administration of several psychological assessments. These included the Trail Making Test (TMT), which was used to evaluate mental flexibility, the Stroop test, which was employed to assess cognitive inhibition, and the letter “P” and animal fluencies (both used following the GREFEX recommendations, France), which were utilized to evaluate information generation. It has been acknowledged that the latter two tasks are particularly sensitive indicators of executive function [33]. The evaluation of planning abilities was conducted using the ROCF copy [31]. In this evaluation, practitioners must exercise particular discernment in regard to the nature of the patient’s responses, as these responses serve as an accurate indicator of the patient’s capacity to formulate an effective response to a given problem [31]. Finally, as previously outlined, the evaluation of working memory was conducted using the backward digit span, as outlined in the Wechsler Adult Intelligence Scale, Fourth Edition (WAIS-IV) [27] and Corsi’s blocks [28]. The evaluation of instrumental functions, encompassing multiple domains, such as language, ideomotor and motor praxis, and visuo-construction, was conducted using the DO 80 test for French patients (ECPA, now Pearson France, Paris, France). The assessment of language (naming capabilities) was facilitated by this instrument [34]. This evaluation method analyzes denomination through image-based abilities. The assessment of praxis is conducted using a specialized battery, such as the Mahieux praxis battery (developed by Mahieux et al., commonly used in neuropsychology in France), which is specifically designed for French patients (Praxes) [35] and ROCF (visuo-constructive praxis) [31]. These instruments are characterized by their sensitivity and the minimal time required for their operation. All the other raw performance scores were converted to mean z scores through comparison with a control population to express a pathological threshold at 1.65 SD of the normal average. The threshold of −1.65 standard deviation (SD) as an indicator of pathology in cognitive tests stems from statistical principles applied to the normal distribution of cognitive scores within a given population. This threshold corresponds to the 5th percentile (approximately 95% of scores fall above it) and is used in various contexts to identify performances that significantly deviate from the expected average [36,37,38,39,40]. The threshold of −1 SD is commonly used in neuropsychology to identify cognitive performance slightly below average, without being classified as pathological [38,39,40,41,42]. In a normal distribution, this threshold corresponds approximately to the 16th percentile, indicating that 16% of the population scores below this level. Such a deviation can signal a relative weakness in a specific cognitive function, while remaining within the boundaries of normal variability. This threshold allows clinicians to detect potential difficulties that, although not pathological, may require attention. It is imperative to exercise discernment in the interpretation of results, taking into account the assessment of fatigue, pain, and depression levels. Thus, to determine indicators of fatigue, mood, and pain states, the Beck Depression Inventory-II (Pearson France, Paris, France) [43] and Visual Analogue Scale (widely used tool) [44,45,46] were utilized at the beginning of the assessment. The Beck Depression Inventory-II (BDI-II) is a widely used self-report instrument designed to measure the severity of depressive symptoms. The scale consists of 21 items, each scored from 0 to 3, with a total score ranging from 0 to 63. Threshold scores help categorize the severity of depression. A total score of 0–13 indicates minimal or no depression, 14–19 suggests mild depression, 20–28 reflects moderate depression, and 29–63 signifies severe depression. These cutoff points are based on normative data and clinical research, providing a standardized approach to assess depressive symptoms and aid in diagnostic and treatment planning. The Visual Analogue Scale (VAS) is a simple and widely used tool for assessing subjective experiences, such as pain, discomfort, or fatigue. It consists of a straight line, typically 10 cm long, with one end labeled “0” (no symptom, e.g., no fatigue) and the other labeled “10” (worst imaginable symptom, e.g., worst fatigue imaginable). The individual marks a point on the line that corresponds to the intensity of their experience. The score is determined by measuring the distance from the “0” point to the mark, providing a value between 0 and 10. For example, the fatigue reliable change index (RCI) was established at 3.47 [47].

### 2.3. Procedure

All patients (*n* = 80) underwent the same cognitive tests in the context of care and the neuropsychological evaluation lasted for about 75 min. The order of testing was the same for all patients: Visual Analogical Scale (VAS) for pain and fatigue, BDI-II, forward digit span, backward digit span, FCSRT, Praxes, TMT A and B, Stroop test, Rey figure (copy), Zazzo’s cancellation test, Rey figure (recall, +3 min), FCSRT (delayed recall, +20 min), “P” fluency, “Animals” fluency, and DO80. All the tests were performed in the same session.

### 2.4. Statistics

Basic statistical analyses were employed to calculate and summarize the results of cognitive tests and clinical scales. Measures such as mean and standard deviation (SD) were used. Comparisons of sociodemographic and clinical data were calculated using a *t*-test.

For intra- and inter-group analyses, a combined approach was employed. Logistic regression models were used to evaluate associations between clinical variables, neuropsychological variables, and diagnostic groups (ME/CFS or not), adjusting for confounding factors, such as mood, pain, and fatigue. Additionally, K-means clustering was applied to explore intra-group variability within the ME/CFS population, identifying homogeneous subgroups based on symptomatic and neuropsychological profiles.

## 3. Results

### 3.1. Depression, Pain, and Fatigue Data

ME/CFS patients had a level of depression above the depression threshold (14 points; mean ± SD: 16.08 ± 9.1). Twenty-seven (68%) had scores equal to or above the depression threshold. The VAS score (mean ± SD) of fatigue was 6.68 ± 2.24 and that of pain was 4.39 ± 2.1.

The level (mean ± SD) of depression in MS patients was 19.05 ± 12.66. Twenty-four (60%) had scores above the depression threshold. The levels (mean ± SD) of fatigue and pain were 5.21 ± 3.04 and 4.43 ± 2.91, respectively.

### 3.2. Cognitive Data

ME/CFS patients exhibited a weak performance in the visual span forward test (mean ± SD: −1.34 ± 0.6), at all the free recalls (−1.18 ± 1.49), and at delayed free recall (−1.49 ± 1.76) of the FCSRT. They also obtained weakened scores in the three-signs condition of Zazzo’s cancellation test (−1.40 ± 1.12) and the Stroop color naming test (1.12 ± 1.83). They were above the pathological threshold in terms of the Stroop word reading test (1.97 ± 3.79) and total delayed recall of the FCSRT (−2.53 ± 5.47; Table 3).

MS patients showed a weak delayed free recall (−1.02 ± 1.22) in the FCSRT. They also showed low scores under the three-signs condition of Zazzo’s cancellation test (−1.02 ± 1.38), the Stroop color naming test (1.24 ± 1.83), and the Stroop word reading test (1.43 ± 2.15; Table 3).

### 3.3. Comparison of ME/CFS and MS Patients

The multivariable analysis (Table 4) evaluated associations between various clinical variables and the probabilities of belonging to the ME/CFS group, while controlling for confounding factors. Clinical variables included measures of fatigue (VAS Fatigue), pain (VAS Pain), and mood (BDI-II), as well as neuropsychological indicators, such as FCSRTDTR and TMT B-A. Confounding factors accounted for included medical treatments received, levels of clinical symptoms at the time of evaluation (fatigue, pain, and mood), and sociodemographic characteristics. The results showed that impaired cognitive performance, particularly a score < −1.65 on FCSRTDTR (OR = 6.12; *p* = 0.02), which is predictive of belonging to the ME/CFS group, and a score ≥ 1.65 on TMT B-A (OR = 0.14; *p* = 0.03), associated with a lower probability of belonging to the CFS group. Additionally, fatigue levels ≥ 3 on VAS Fatigue emerged as a major predictive factor (OR = 7.34; *p* = 0.03). Conversely, the impact of treatments and pain levels appeared less significant. Other neuropsychological tests were not included in the final results of the analysis due to the lack of significant differences in performance between the ME/CFS and MS groups. This suggested that these tests may evaluate cognitive functions that are similarly affected in both conditions.

### 3.4. Neuropsychological Stratification of ME/CFS Patients

**Figure 1:**
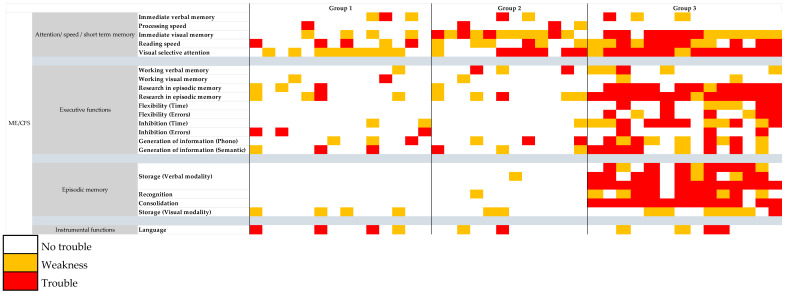
Neuropsychological stratification of ME/CFS patients.

On the basis of the neuropsychological finding on the heterogeneity of cognitive impairment in ME/CFS [48,49,50,51,52,53], we managed to classify ME/CFS patients into three groups, forming a continuum with a gradient of severity going from group 1 to group 3 (Figure 1): (i) group 1 (15/40): very mild cognitive impairment, without a pathological score, but with a slowdown in reading speed (score > +1 SD for the Stroop words condition) for 33% of them; (ii) group 2 (12/40): moderate cognitive impairment with a minimum weakness (score < –1 SD) in selective attention in visual entry, immediate visual memory, and reading speed; (iii) group 3 (14/40): definite cognitive impairment with a deficit (score < −1.65 SD) in selective visual attention, reading speed, and the capacities for storage and consolidation in episodic verbal memory, as well as a weakness in immediate visual-sequential memory and executive functions (mental flexibility, cognitive inhibition, and information generation).

In the same way, the logistic regression analysis identified the FCSRTDTR as a strong predictor of group classification, with a score < −1.65 significantly increasing the odds of belonging to the ME/CFS group (OR = 6.12; *p* = 0.02). This finding aligns with previous research highlighting heterogeneity in cognitive impairments in ME/CFS populations. Specifically, the FCSRTDTR plays a pivotal role in distinguishing among the three severity-based cognitive impairment groups identified in previous studies. In addition, K-means clustering was conducted to explore symptomatic and neuropsychological variations within the population diagnosed with ME/CFS (Table 5). This analysis revealed two distinct subgroups within the population diagnosed with chronic fatigue syndrome (CFS), highlighting differences in symptomatic and neuropsychological profiles. The first group (Cluster 1, *n* = 27) exhibited pain (VAS Pain = 4.12 ± 2.09), fatigue (VAS Fatigue = 6.44 ± 2.17), and mild depressive symptoms (BDI-II = 15.04 ± 8.48). Neuropsychologically, their cognitive performance was close to normal, as indicated by scores such as FCSRTDTR (0.31 ± 0.47) and TMT B-A (−0.37 ± 0.64), suggesting preserved memory and cognitive flexibility. This group also demonstrated good inhibitory capacity (Stroop interference = 0.15 ± 0.68) and a normal BMI (20.11 ± 3.41).

The second group (Cluster 2, *n* = 13) displayed more pronounced symptoms, including pain (EVA Pain = 4.93 ± 2.10), fatigue (VAS Fatigue = 7.18 ± 2.40), and depressive symptoms (BDI-II = 18.23 ± 10.29). Their neuropsychological profile was marked by significant impairments in episodic memory (FCSRTDTR = –8.43 ± 6.39), cognitive flexibility (TMT B-A = 1.45 ± 1.15), and inhibition (Stroop interference = 2.51 ± 1.64). Additionally, this group had a higher BMI (28.77 ± 3.68)

## 4. Discussion

The present study drew parallels and distinctions between the cognitive impairments associated with ME/CFS and MS. The neuropsychological battery employed for the routine evaluation of patients was meticulously designed as a compromise to minimize the duration of neuropsychological evaluation, thereby limiting the impact of fatigue, while striving to evaluate various cognitive functions in an efficient manner. Notably, it excluded tests such as the PASAT [54], CVLT [55], and the SDMT [56]. These tools are frequently utilized in the neuropsychological evaluation of patients with MS. They are also employed, on occasion, to assess the cognitive capacities of individuals with ME/CFS [57]. The decision to utilize the FCSRT was predicated on the premise that we had previously indicated the existence of potential disorders that could potentially impact several of these processes. Consequently, it is imperative to examine the integrity of the encoding phase [58]. Indeed, the degree to which information is encoded is contingent upon the level of attention allocated to its memorization. A dearth of attention on the information in question has been shown to adversely affect the quality of storage as well as the subsequent retrieval of that information [59]. The selection of tests for the evaluation of episodic memory was made with the objective of regulating the quality of encoding by reducing the effect of potential variations in attention levels. The FCSRT imposes and controls the encoding mode, thereby limiting the potential bias of attention that may hinder the processing of the information to be memorized [29]. It must be acknowledged that this may impose limitations on the ability to make comparisons between studies. Nevertheless, the battery demonstrated its reliability in assessing the primary cognitive domains, while also requiring the least time, a crucial consideration when evaluating patients with ME/CFS.

With regard to ME/CFS, our classification confirmed the existence of inter-patient heterogeneity, within a well-defined syndromic framework excluding any impairment of instrumental functions. If the groups differed in functions, such as executive functions, selective attention to visual input, or episodic memory, there was a common symptomatologic core in that a weakness at least in immediate visual-sequential memory was present. There was, therefore, a continuum in terms of symptomatology with a gradient of severity from group 1 to group 3 and a disorder common to all patients, concerning immediate visual memory (the auditory–verbal modality was apparently preserved). The idea that this symptomatology could be specific to ME/CFS was supported by the frequency of onset of symptoms, which was high in ME/CFS patients and significantly more expressed than in MS patients with regard to the common disorder affecting visual immediate memory but also consolidation disorders. This information, which still needs to be confirmed, is not anecdotal. Indeed, the visuospatial memory is believed to be supported by the fronto-temporo-parietal and occipital regions [60,61,62], and functional brain imaging uncovered hypometabolism of posterior associative regions [63,64]. Put together, these data suggested the implication of several specific cortical areas in the cognitive deficit of ME/CFS. The clustering analysis revealed that group 2 presented higher levels of fatigue and pain compared to group 1, aligning with the diagnostic criteria of ME/CFS. While an interpretation could attribute the cognitive impairments in group 2 to these factors, an alternative perspective emerged. It is striking to observe that the exacerbation of core ME/CFS symptoms, such as fatigue and pain, was consistently mirrored by a worsening of cognitive impairments. This pattern suggests a cohesive aggravation across all dimensions of the disease. As fatigue and pain intensified, so too did deficits in memory, cognitive flexibility, and inhibitory control and attention, reflecting an intertwined progression of both physical and cognitive aspects of the disorder. These findings underscore the systemic nature of ME/CFS, where worsening symptomatology spans multiple domains [6,65,66,67]. Similarly, an alternative analysis might attribute cognitive impairments in ME/CFS primarily to depressive symptoms, given their prevalence in this population. However, this perspective overlooks the broader pattern of symptom aggravation across multiple domains. The clustering analysis showed that as fatigue and pain intensified, there was a concomitant decline in cognitive performance, suggesting that these impairments were not solely triggered by depression. Conversely, the overall worsening of ME/CFS symptomatology can significantly impact mood and quality of life. This was corroborated by the increased BMI observed in group 2, potentially linked to reduced mobility and lifestyle changes. These interconnected factors underscore the systemic nature of ME/CFS and the complex interplay between physical, cognitive, and emotional domains.

One cognitive symptom, which appeared common to ME/CFS and MS patients, was the slowing down of reading speed. This element was assessed by the fact that on the one hand, neither of the two groups, ME/CFS and MS, showed a slowing down at TMT A, but on the other hand, both groups showed a slowdown in the color and word conditions of the Stroop test. However, the Stroop test, due to its sensitivity to eye saccades [68], tends to replicate the conditions in a reading situation [69,70,71]. Patients with multiple sclerosis are likely to present a disorder affecting eye movements [72,73], which seems to be able to impact the level of performance of tasks related to visual inputs, regardless of whether it concerns selective attention or reading speed [74]. ME/CFS patients showed impairment in the Stroop test, an outcome that could be attributed at least in part to eye movement dysfunctions. Interestingly, it was shown that continuous pursuit activity (accurately tracking a moving object), even for a short time period, could reveal dysfunctional eye movement behavior in ME/CFS patients [75], suggesting that oculometry could probably contribute to the diagnosis of ME/CFS.

Difficulties in episodic memory were also encountered in both pathologies, with a common denominator of a deficit in the executive component of episodic memory, namely, the ability to recover information stored or consolidated in episodic memory [33]. This deficit is described in multiple sclerosis [19] and possibly related to white matter involvement, playing an important role in the efficiency of executive functions [76]. Such a deficit is less described in the context of ME/CFS, for which there is more evidence of a disorder concerning the encoding phase [29,58] and consolidation [77]. However, memory retrieval difficulties in the ME/CFS patients included in our study could not be investigated due to their storage capacities being affected, as well as consolidation difficulties. These symptoms were significantly less likely to be found in MS patients. The difference in memory profile suggests the presence of an organic etiology contributing to memory impairments observed in individuals diagnosed with ME/CFS. In effect, the consolidation and storage processes are underpinned by the internal temporal regions and, more particularly, by the hippocampi [78,79], included in the circuit of Papez [79]. A deficit of these processes could be due to an alteration involving these structures, as has been described in other pathologies, such as Alzheimer’s disease [80] or temporal epilepsy [81]. Moreover, cellular and metabolic abnormalities have been described by functional imaging in ME/CFS [63,64,82,83], tending to raise the question of a possible correlation between disorders affecting episodic memory and a dysfunction affecting internal temporal structures.

The mechanisms underlying cognitive dysfunction in ME/CFS remain to be elucidated. Conventional imaging procedures yield normal results and are primarily employed for differential diagnoses. Neuroinflammation can be visualized through positron emission tomography (PET) brain imaging using a radioligand for translocator protein (TSPO), a protein belonging to the mitochondrial permeability transition pore (MPTP) complex and produced when microglia are activated [67]. This approach demonstrated a correlation between cognitive impairment in patients with myalgic encephalomyelitis/chronic fatigue syndrome (ME/CFS) and positron emission tomography (PET) signals, particularly in the region between the mid-pons and thalamus [82], which support the hypothesis of neuroinflammation in myalgic encephalomyelitis/chronic fatigue syndrome (ME/CFS).

Furthermore, this study has several strengths, particularly in its rigorous methodology and comparative approach between ME/CFS and MS patients. By applying strict inclusion and exclusion criteria, we ensured the homogeneity of the sample, reducing potential confounding factors, such as neurological comorbidities or treatment effects. Furthermore, the use of both multivariate analyses (logistic regression) and unsupervised clustering (K-means) provided complementary insights into the cognitive and symptomatic heterogeneity within the ME/CFS population. The comprehensive neuropsychological assessment allowed us to identify specific cognitive impairments in ME/CFS, distinguishing them from those observed in MS patients. Additionally, the study contributes to the field by highlighting that cognitive dysfunction in ME/CFS is not merely a consequence of fatigue, pain, or depression, reinforcing the hypothesis of an intrinsic neurocognitive alteration in this condition.

However, this study also has limitations. The sample size was moderate, which may limit the generalizability of our findings to the broader ME/CFS and MS populations. Additionally, while we controlled for major confounders, other factors, such as sleep disturbances, autonomic dysfunction, or subtle mood fluctuations, were not explicitly accounted for in the analysis and could influence cognitive performance. Another limitation is the cross-sectional design, which did not allow us to assess the evolution of cognitive impairments over time or their potential responsiveness to therapeutic interventions. Finally, while our statistical approach was robust, alternative multivariate methods, such as principal component analysis (PCA) or partial least squares regression (PLSR), could further explore complex relationships between cognitive, symptomatic, and biological parameters, providing additional insights in future studies. Despite these limitations, we suppose that this study represents an important step toward a better understanding of ME/CFS-related cognitive impairments, offering potential implications for both diagnostic refinement and targeted cognitive rehabilitation strategies. Future research should aim to validate these findings in larger, longitudinal cohorts, integrating multimodal approaches, such as neuroimaging and electrophysiological markers, to further elucidate the neurobiological underpinnings of cognitive dysfunction in ME/CFS.

Based on the findings of this study, several practical recommendations can be made to improve the clinical management of ME/CFS, particularly regarding cognitive impairments. Given that ME/CFS patients exhibit specific deficits in episodic memory retrieval, selective visual attention, reading speed, and consolidation processes, targeted cognitive rehabilitation strategies should be implemented. Neuropsychological interventions should focus on enhancing memory encoding and retrieval through structured cognitive training programs, particularly those involving verbal learning strategies and working memory exercises. Additionally, given the strong impact of attention deficits, cognitive remediation programs incorporating attentional control training may help improve daily functioning.

From a clinical perspective, multidisciplinary care should be emphasized, integrating neurologists, neuropsychologists, physiotherapists, and occupational therapists to address the multifaceted aspects of ME/CFS. Given the observed absence of correlation between cognitive impairments and fatigue, pain, or depression, cognitive dysfunction should be assessed independently rather than being attributed solely to general symptoms. This highlights the necessity of systematic neuropsychological evaluations in ME/CFS patients reporting cognitive complaints, as cognitive impairment may not always align with subjective fatigue severity.

Furthermore, given the heterogeneity of cognitive deficits revealed in this study, a personalized approach to treatment is crucial. Patients presenting with predominant memory impairments may benefit from mnemonic techniques and compensatory strategies, while those with attentional dysfunctions may require task-switching training and executive function support. In addition, considering the potential role of cortico-subcortical network dysfunctions, non-invasive neuromodulation techniques, such as transcranial direct current stimulation (tDCS) [84,85,86,87,88] or neurofeedback training [89,90,91,92], targeting prefrontal and central regions could be explored as adjunct therapies.

Finally, clinicians should provide lifestyle recommendations to optimize cognitive performance in ME/CFS patients. These include sleep hygiene strategies, physical activity adapted to post-exertional malaise, and dietary interventions targeting neuroinflammation and oxidative stress [93,94,95,96]. Future research should investigate the efficacy of combined cognitive rehabilitation and neuromodulatory approaches to develop personalized, evidence-based interventions aimed at improving cognitive function and overall quality of life in ME/CFS patients.

## 5. Conclusions

In conclusion, the present study highlighted both similarities and differences in the cognitive phenotypes of ME/CFS and MS. Cognitive impairments in ME/CFS patients may be similar to or even more severe and frequent than those observed in MS patients. Neuropsychological stratification suggested that cognitive symptomatology in ME/CFS varies among individuals but remains confined within a restricted syndromic framework, excluding disturbances in instrumental functions. While certain impairments, such as visual immediate memory deficits, appear to be common to most patients, the presence of additional symptoms seems to depend on the severity of cognitive dysfunction.

Further studies will be necessary to validate these findings in larger cohorts and correlate them with functional brain imaging data. Advanced neuroimaging techniques, including innovative functional imaging procedures and oculometry, should be integrated into cognitive assessments. This step is crucial for identifying the organic mechanisms underlying cognitive dysfunction, thus contributing to the recognition of ME/CFS as a distinct neurological disease rather than a syndrome.

Moreover, the 2024 report from the ME/CFS Research Roadmap Working Group [97] emphasized the importance of a multidisciplinary approach that incorporates neurology, immunology, metabolism, and genetics to refine both diagnostic and therapeutic strategies. As ME/CFS is a multifactorial disorder involving immune dysfunction, neuroinflammation, metabolic disturbances, and genetic predispositions, future research must prioritize integrated methodologies, such as multi-omics profiling, neurophysiological assessments, and personalized treatment strategies. The convergence of biomedical, technological, and clinical expertise will be essential to enhancing diagnostic precision, developing targeted therapies, and ultimately, improving patient outcomes.

## Figures and Tables

**Table 1:** Sociodemographic and clinical characteristics of ME/CFS and MS patients.

Variable	ME/CFS (*n* = 40)	MS (*n* = 40)	*p*-Value
Sociodemographic variables			
Age (years)	46.9 ± 12.9	45.7 ± 11.6	0.63
Sex (% female)	55%	68%	0.78
Education (median (range))	6 (3–7)	6 (2–7)	0.67
Clinical variables			
Disease duration (years)	13.1 ± 5.8	10.8 ± 8.3	0.62
Fatigue (VAS)	6.68 ± 2.24	5.21 ± 3.04	0.02 *
Pain (VAS)	4.39 ± 2.1	4.43 ± 2.91	0.94
BDI-II score	16.0 ± 9.1	19.05 ± 12.7	0.23
BMI	22.9 ± 5.3	26 ± 5.9	0.26
% on mood treatments	55%	37.5%	0.32

(*) *p* < 0.05, indicating a statistically significant difference between groups. BDI-II: Beck Depression Iinventory (2nd edition), VAS: Visual Analogue Scale, BMI: Body Mass Index.

**Table 2:** Neuropsychological assessment of patients with chronic fatigue and cognitive complaints.

Domain	Tests, Scales
Pain, fatigue, depression	BDI-II (Depression), VAS (Pain), VAS (Fatigue)
Memories	Forward digit span, FCSRT, ROCF recall (3 min)
Executive functions and attention	Backward digit span, FCSRT, TMT A and B, Stroop,
	P and “Animal” fluencies, ROCF (copy), Zazzo’s cancellation test
Instrumental functions	Praxes, Boston naming test, or DO 80

BDI-II: Beck Depression Inventory (2nd edition), VAS: Visual Analogue Scale, FCSRT: Free and Cued Selective Reminding Test, ROCF: Rey-Osterrieth Complex Figure, TMT: Trail Making Test, DO: Dénomination orale.

**Table 3:** Results by domain for ME/CFS and MS populations.

		ME/CFS		MS	
Domain	Test	*N*	Mean ± SD	*N*	Mean ± SD
Short-Term Memory	Verbal span forward	40	−0.45 ± 0.77	40	−0.32 ± 1.01
	Verbal span backward	40	−0.38 ± 1.03	40	−0.64 ± 0.89
	Visual span forward	40	−1.34 ± 0.60	40	−0.91 ± 1.01
	Visual span backward	40	−0.04 ± 0.81	40	−0.01 ± 1.1
Long-Term Memory	FCSRT Imm	40	−0.90 ± 1.95	40	−0.77 ± 1.84
	FCSRT TR1	40	−0.51 ± 1.58	40	0.09 ± 0.89
	FCSRT TR2	40	−0.46 ± 1.56	40	−0.34 ± 1.64
	FCSRT TR3	40	−1.09 ± 2.94	40	−0.35 ± 2.07
	FCSRT Tot 3 TR	40	0.29 ± 4.94	40	1.67 ± 3.9
	FCSRT Rec	40	−0.98 ± 3.56	40	−0.65 ± 2.42
	FCSRT DTR	40	−2.53 ± 5.47	40	−0.61 ± 2.53
	ROCF Recall	40	−0.38 ± 1.05	40	−0.62 ± 1.03
Executive Functions	FCSRT 3FR	40	−1.18 ± 1.49	40	−0.95 ± 1.11
	FCSRT DFR	40	−1.49 ± 1.76	40	−1.02 ± 1.22
	TMT A	40	−0.15 ± 1.08	40	0.26 ± 1.1
	TMT B-A	40	0.22 ± 1.20	40	0.87 ± 2.44
	TMT B-A Err	40	0.16 ± 1.19	40	0.53 ± 1.81
	Stroop C	40	1.12 ± 1.83	40	1.24 ± 1.83
	Stroop W	40	1.97 ± 3.79	40	1.43 ± 2.15
	Stroop Interference	40	0.92 ± 1.55	40	1.02 ± 1.14
	Stroop I-D	40	0.50 ± 1.15	40	0.57 ± 1.17
	Stroop I-D err	40	0.48 ± 2.38	40	−0.42 ± 1.90
	P Fluencies	40	−0.18 ± 1.72	40	−0.51 ± 1.07
	Anim Fluencies	40	−0.45 ± 1.53	40	−0.64 ± 1.10
Attention	Zazzo 3 signs	40	−1.40 ± 1.12	40	−1.02 ± 1.38
Language	DO 80	40	−0.35 ± 1.49	40	−1.02 ± 3.01
Visuo-construction	ROCF Copy	40	0.1 ± 0.81	40	−0.26 ± 1.24
Mood	BDI II	40	16.08 ± 9.1	40	19.05 ± 12.66
Pain	VAS Pain	40	4.39 ± 2.1	40	4.43 ± 2.91
Fatigue	VAS Fatigue	40	6.68 ± 2.24	40	5.21 ± 3.04

FCSRT: Free and Cued Selective Reminding Test; TR: total recall; DTR: delayed total recall; FR: free recall; DFR: delayed free recall; Rec: recognition; ROCF: Rey-Osterrieth Complex Figure; TMT: Trail Making Test; C: colors; W: words; I-D: interference—denomination; err: error; Anim: animals; DO: Dénomination Orale; BDI: Beck Depression Inventory; VAS: Visual Analogue Scale.

**Table 4:** Multivariable analysis with adjustments for mood, pain, fatigue, and treatment.

		*N* = 80	
		Odds Ratio (CI 95%)	*p*-Value
FCSRTDTR	≥−1.65	1 (ref.)	
	<−1.65	6.12 (1.34; 27.95)	0.02
TMTBA	<1.65	1 (ref.)	
	≥1.65	0.14 (0.02; 0.78)	0.03
BDI-II	[0;13]	1 (ref.)	
	[13;28]	1.56 (0.48; 5.08)	0.46
	[28;63]	0.17 (0.03; 0.98)	0.047
VAS Pain	<3	1 (ref.)	
	≥3	1.44 (0.45; 4.68)	0.54
VAS Fatigue	<3	1 (ref.)	
	≥3	7.34 (1.19; 45.29)	0.03
Treatment	None	1 (ref.)	
	Pregabalin/Amitriptylin/Duloxetin/Escitalopram/Paroxetin	1.68 (0.58; 4.84)	0.34

CI 95%: 95% confidence interval.

**Table 5:** Results of the clustering with K-means in the population of ME/CFS.

		Cluster 1,		Cluster 2,	
		*N* = 27,		*N* = 13,	
		Mean (±SD)	Median (IQR)	Mean (±SD)	Median (IQR)
Sociodemographic and clinical data					
Age		48.11 (±12.81)	51.00 (39.00; 60.00)	44.38 (±13.18)	45.00 (43.00; 49.00)
Delta		12.25 (±6.12)	12.00 (9.50; 17.00)	15.75 (±4.03)	16.00 (12.50; 19.00)
BMI		20.11 (±3.41)	19.00 (18.00; 22.00)	28.77 (±3.68)	28.00 (26.00; 32.00)
Neuropsychological testing					
Verbal forward		−0.33 (±0.84)	−0.45 (−0.75; 0.32)	−0.70 (±0.55)	−0.53 (−0.92; −0.15)
Verbal backward		−0.18 (±1.12)	−0.25 (−0.75; 0.31)	−0.79 (±0.68)	−0.69 (−1.23; −0.46)
Visual forward		−1.20 (±0.55)	−1.27 (−1.50; −0.71)	−1.63 (±0.60)	−1.60 (−1.95; −1.22)
Visual backward		0.03 (±0.88)	−0.05 (−0.34; 0.62)	−0.20 (±0.65)	−0.20 (−0.45; 0.18)
FCSRT Imm		−0.70 (±1.80)	0.43 (−1.60; 0.43)	−1.29 (±2.24)	−0.56 (−1.67; 0.40)
FCSRT 3FR		−0.45 (±0.98)	−0.52 (−0.93; 0.31)	−2.69 (±1.19)	−2.66 (−3.20; −2.30)
FCSRT TR1		0.13 (±0.82)	0.12 (0.06; 0.69)	−1.84 (±1.94)	−1.50 (−2.82; 0.06)
FCSRT TR2		0.28 (±0.63)	0.58 (0.11; 0.63)	−1.99 (±1.80)	−1.88 (−1.92; −0.63)
FCSRT TR3		0.23 (±0.52)	0.50 (0.20; 0.56)	−3.83 (±3.94)	−2.78 (−4.50; −1.80)
FCSRT Tot 3 TR		2.52 (±2.41)	3.00 (2.00; 4.00)	−4.35 (±5.68)	−4.00 (−5.00; −1.00)
FCSRT Rec		0.31 (±0.30)	0.43 (0.17; 0.43)	−3.66 (±5.43)	−1.50 (−3.17; −1.50)
FCSRT DFR		−0.58 (±1.04)	−0.50 (−1.19; 0.07)	−3.39 (±1.41)	−3.28 (−4.94; −2.17)
FCSRT DTR		0.31 (±0.47)	0.38 (0.33; 0.38)	−8.43 (±6.39)	−6.33 (−9.67; −3.38)
Rey Copy		0.08 (±0.81)	0.40 (−0.30; 0.84)	0.13 (±0.84)	0.40 (0.00; 0.84)
Rey Recall		−0.09 (±0.96)	−0.08 (−1.03; 0.38)	−0.99 (±0.99)	−0.96 (−1.23; −0.56)
TMT A		−0.05 (±1.23)	−0.42 (−0.79; 0.25)	−0.35 (±0.68)	−0.21 (−0.79; 0.00)
TMT B-A		−0.37 (±0.64)	−0.59 (−0.90; 0.16)	1.45 (±1.15)	1.05 (0.91; 1.86)
TMT B-A err		−0.24 (±0.62)	−0.30 (−0.42; −0.27)	0.98 (±1.64)	−0.27 (−0.38; 1.70)
Zazzo 3 signs		−0.96 (±1.00)	−1.20 (−1.71; 0.09)	−2.32 (±0.75)	−2.48 (−2.82; −1.71)
Stroop C		0.41 (±0.87)	0.44 (−0.32; 1.23)	2.59 (±2.40)	1.78 (1.31; 3.58)
Stroop W		0.73 (±1.40)	0.50 (−0.33; 1.50)	4.54 (±5.65)	2.80 (1.30; 3.67)
Stroop interference		0.15 (±0.68)	0.22 (−0.31; 0.66)	2.51 (±1.64)	2.67 (1.41; 2.89)
Stroop I-D		−0.03 (±0.76)	0.00 (−0.67; 0.57)	1.60 (±1.06)	1.59 (1.17; 2.06)
Stroop I-D err		0.21 (±1.76)	−0.36 (−0.37; −0.25)	1.03 (±3.35)	−0.36 (−0.37; −0.25)
P fluencies		0.37 (±1.76)	0.11 (−0.91; 1.39)	−1.33 (±0.87)	−1.52 (−1.85; −0.83)
Anim fluencies		0.09 (±1.40)	0.10 (−0.93; 0.84)	−1.55 (±1.20)	−1.54 (−2.40; −0.75)
DO 80		−0.34 (±1.66)	0.32 (−0.97; 0.70)	−0.36 (±1.10)	−0.04 (−1.30; 0.70)
Mood, Pain, Fatigue					
BDI-II		15.04 (±8.48)	13.00 (10.00; 22.00)	18.23 (±10.29)	17.00 (12.00; 21.00)
VAS Pain		4.12 (±2.09)	4.60 (2.80; 5.40)	4.93 (±2.10)	5.00 (4.30; 5.30)
VAS Fatigue		6.44 (±2.17)	7.00 (5.20; 8.00)	7.18 (±2.40)	7.40 (5.20; 10.00)
Sociodemographic and clinical data					
Gender	1	18 (66.67%)		0 (0.00%)	
	2	9 (33.33%)		13 (100.00%)	
Pathology	CFS	27 (100.00%)		13 (100.00%)	
Type	1	14 (51.85%)		0 (0.00%)	
	2	12 (44.44%)		0 (0.00%)	
	3	1 (3.70%)		13 (100.00%)	
SCL	3	4 (14.81%)		1 (7.69%)	
	4	5 (18.52%)		2 (15.38%)	
	5	4 (14.81%)		3 (23.08%)	
	6	6 (22.22%)		4 (30.77%)	
	7	8 (29.63%)		3 (23.08%)	
Laterality	D	25 (92.59%)		13 (100.00%)	
	G	2 (7.41%)		0 (0.00%)	
Treatment	Amitriptylin	5 (27.78%)		3 (75.00%)	
	Duloxetin	8 (44.44%)		1 (25.00%)	
	Pregabalin	5 (27.78%)		0 (0.00%)	

## Data Availability

The raw data supporting the conclusions of this article will be made available by the authors on request.

## Supplementary Materials

The following supporting information can be downloaded at: https://www.mdpi.com/article/10.3390/diagnostics15040487/s1, Table S1: MRI-Based Diagnostic Criteria for Multiple Sclerosis (MS): Comparison of Key Guidelines.
