# Supplementary material for: Comparative Study Between Cognitive Phenotypes of Myalgic Encephalomyelitis/Chronic Fatigue Syndrome and Multiple Sclerosis
Source: Diagnostics (Basel). 2025 Feb 17;15(4):487. doi: 10.3390/diagnostics15040487 (PMC11854609; doi:10.3390/diagnostics15040487)
Supplement: Supplementary file 1 [file diagnostics-15-00487-s001.zip › diagnostics-3435668-supplementary.pdf]

Table S1. MRI-Based Diagnostic Criteria for Multiple Sclerosis (MS): Comparison of Key Guidelines

Diagnostic Criteria for MS (MRI)	McDonald et al., 2001	Thompson et al., 2018 (McDonald Revision)	Barkhof et al., 2003
**Dissemination in Space (DIS)**	At least two lesions in typical locations (juxtacortical, periventricular, infratentorial, spinal cord).	Simplified and improved accuracy with advanced MRI sequences while maintaining DIS definition.	Validation of DIS criteria with correlation between MRI findings and clinical signs.
**Dissemination in Time (DIT)**	Lesions at different time points (gadolinium-enhancing and non-enhancing lesions on a single MRI).	Asymptomatic infratentorial lesion confirmed by a new lesion on follow-up MRI. Early detection focus.	Diagnostic value of both new and old lesions to confirm DIT.
**Characteristic Lesions**	Periventricular, juxtacortical, infratentorial, and spinal cord lesions (brain and spinal MRI recommended).	No change in lesion criteria; better identification of cortical and infratentorial lesions with advanced imaging techniques.	Correlation between clinical outcomes and response to interferon β1a treatment.
**Use of MRI for Monitoring**	Recommended to show disease progression and establish early diagnosis.	Extended role of MRI in follow-up and differential diagnosis.	Confirmed therapeutic response to interferon β1a based on MRI changes.

Information noted at the end of each letter sent to patients following their consultation or hospitalization:

Information for patients: The AP-HP informs you that your personal data, collected as part of the administrative management of your care at the hospital, may be used for research purposes in the field of health, under the responsibility of the AP-HP. In particular, a Health Data Warehouse (EDS) has been set up to enable non-interventional research to be carried out on data, clinical trial feasibility studies and hospital activity management studies. For more information about each research project, the data used, the recipients of the data, data retention periods and how to exercise your rights, please consult the EDS information portal at the following address http://recherche.aphp.fr/eds. To object to the use of your data for research purposes, you can contact the users' office or the director of the hospital where you were treated, or fill in the electronic objection form available at the following address http://recherche.aphp.fr/eds/droit-opposition.
